# A mitochondrial genome assembly of the opal chimaera, *Chimaera opalescens* Luchetti, Iglésias et Sellos 2011, using PacBio HiFi long reads

**DOI:** 10.1080/23802359.2022.2044403

**Published:** 2022-03-02

**Authors:** Nair Vilas-Arrondo, André Gomes-dos-Santos, Montse Pérez, Francisco Baldó, Ana Veríssimo, Diana Catarino, André M. Machado, Esther Román-Marcote, Rafael Bañón, Elsa Froufe, L. Filipe C. Castro

**Affiliations:** a AQUACOV, Instituto Español de Oceanografía (IEO, CSIC), Centro Oceanográfico de Vigo, Vigo, Spain; b UVIGO, PhD Program “Marine Science, Technology and Management” (Do*MAR), Faculty of Biology, University of Vigo, Vigo, Spain; c CIIMAR/CIMAR – Interdisciplinary Centre of Marine and Environmental Research, University of Porto, Matosinhos, Portugal; d Department of Biology, Faculty of Sciences, University of Porto, Porto, Portugal; e Instituto Español de Oceanografía (IEO, CSIC), Centro Oceanográfico de Cádiz, Cádiz, Spain; f BIOPOLIS Program in Genomics, Biodiversity and Land Planning, CIBIO, Vairão, Portugal; g Ocean Sciences Institute - Okeanos, Universidade dos Açores, Horta, Portugal; h Servizo de Planificación, Consellería do Mar, Xunta de Galicia, Santiago de Compostela, Spain; BIOPESLE, Instituto Español de Oceanografía (IEO, CSIC), Centro Oceanográfico de Vigo, Vigo, Spain; i Grupo de Estudos do Medio Mariño (GEMM), Ribeira, Spain

**Keywords:** Chondrichthyes, Chimaeridae, short-nosed chimeras, PacBio

## Abstract

Chondrichthyans (sharks, rays and chimeras) are a fascinating and highly vulnerable group of early branching gnathostomes. However, they remain comparatively poorly sampled from the point of view of molecular resources, with deep water taxa being particularly data deficient. The development of long-read sequencing technologies enables the analysis of phylogenetic relationships through a precise and reliable assembly of complete mtDNA genomes. The sequencing and characterization of the complete mitogenome of the opal chimera *Chimera opalescens* Luchetti, Iglésias et Sellos 2011, using the long-read technique PacBio HiFi is presented. The entire mitogenome was 23,411 bp long and shows the same overall content, i.e. 13 protein-coding genes, 22 transfer RNA and 2 ribosomal RNA genes, as all other examined Chondrichthyan mitogenomes. Phylogenetic reconstructions using all available Chondrichthyan mitogenomes, including 11 Holocephali (chimeras and ratfishes), places *C. opalescens* within the Chimaeridae family. Furthermore, the results reinforce previous findings, showing the genus *Chimera* as paraphyletic and thus highlighting the need to expand molecular approaches in this group of cartilaginous fishes.

Chondrichthyans are a monophyletic clade with two sister taxa, the Elasmobranchii (sharks and rays) and Holocephali (chimeras). Their *K-*selective reproductive traits, such as large body size and slow growth rate (Calis et al. 2005; Dagit et al. 2007; Kraft et al. 2020; Kousteni 2021), make them vulnerable to human-mediated threats such as overfishing, particularly elasmobranchs (Cavanagh and Gibson 2007; Dulvy et al. 2014; Oliver et al. 2015; Dulvy and Trebilco 2018). Chimaerid are also a frequent by-catch of deep-water fisheries (Blasdale and Newton, 1998; Moura et al., 2004; Catarino et al. 2020). Holocephalans comprise a single surviving order, the Chimaeriformes (Wyffels et al. 2014). The described species are allocated into three different families: Callorhinchidae, Rhinochimaeridae and Chimaeridae (Weigmann 2016). Furthermore, the family Chimaeridae only includes two genera: *Chimera* Linnaeus 1758 and *Hydrolagus* Gill 1862 (Weigmann 2016). Recently, several new species have been described (e.g. Iglésias et al. 2022), including *Chimera opalescens* Luchetti et al. 2011 from deep-sea assemblages (Luchetti et al. 2011). This species is widely distributed in the eastern Atlantic, with records in the British Isles and France (Luchetti et al. 2011), on the banks of Greenland, Gorringe and Galicia (Bañon et al. 2016; Luchetti et al. 2011; Vieira and Cunha 2014), Madeira, northwestern slopes of Africa (Freitas et al. 2017) and Azores (Catarino et al. 2020). The species is listed has Least Concern (LC) according with the Red List of Threatened species of the IUCN (https://www.iucnredlist.org/species/18901743/48862329). However, previous records of *C. opalescens* were erroneously classified as *Chimera monstrosa* (Luchetti et al. 2011; Catarino et al. 2020), due to the similar morphology (Luchetti et al. 2011; Didier et al. 2012; Freitas et al. 2017). This type of problems highlights the critical importance of molecular approaches to support species identification. In this context, the development of long-read sequencing technology has been instrumental, since it allows phylogenetic analysis utilizing complete mtDNA genomes (Satoh et al. 2016; Formenti et al. 2021).

A female of *C. opalescens* of 665 mm in total length was captured on 14 October 2020 in the Porcupine Bank (NE Atlantic; Lat:51.1731, Long:-13.5604) at 1037 m depth during the Bottom Trawl Survey PORCUPINE 2020 carried out by the Spanish Institute of Oceanography (IEO, CSIC). Morphological identification was performed onboard, the specimen was frozen, and a muscle tissue sample was stored in absolute ethanol. The specimen is stored at the Spanish Institute of Oceanography in Vigo, with the code voucher C.OPLSCENS_1_P20 (Nair Vilas-Arrondo, nair_vilasarrondo@hotmail.com). The muscle sample is stored at the DNA bank of CIIMAR – Interdisciplinary Center of Marine and Environmental Research with the same voucher code. A small section of the muscle tissue was sent to the Brigham Young University DNA Sequencing Center (BYU), where genomic DNA extraction and whole genome PacBio HiFi library preparation and sequencing were performed, following the manufacturer’s recommendations (Pacific Biosciences; https://www.pacb.com/wp-content/uploads/Procedure-Checklist-Preparing-HiFi-SMRTbell-Libraries-using-SMRTbell-Express-Template-Prep-Kit-2.0.pdf). This work has been approved by the CIIMAR ethical committee and by CIIMAR Managing Animal Welfare Body (ORBEA) according to the European Union Directive 2010/63/EU.

The mitochondrial DNA PacBio HiFi (mtDNA PB) reads were filtered by blast search (Altschul et al. 1990) against a local built Chondrichthyans mitogenome database and after error corrected using Hifiasm (v.0.13-r308; Cheng et al. 2021; Parameters: –write-ec). Subsequently, all reads greater than 20,000 bp were selected and used to perform genome assembly in Unicycler (v.0.4.8.; Parameters: Defaults; Wick et al. 2017) a software optimized for circular genome assemblies.

Gene annotation was performed using MITOS2 webserver (Bernt et al. 2013) and validated by manual comparison with other chimaerids available at NCBI. For the phylogenetic analysis, all available Chondrichthyan mitogenomes were retrieved from the GenBank (https://www.ncbi.nlm.nih.gov/genbank/, accession date 01/03/2021). Individual alignments for the 13 protein-coding genes (PCG) were produced using MAFFT v7.453 (Katoh and Standley 2013) and concatenated using FASconCAT-G (https://github.com/PatrickKueck/FASconCAT-G; final length: 11,431bp). The partition-scheme and the evolutionary best models that fit those schemes and Maximum Likelihood (ML) phylogenetic inference were produced in IQ-TREE (v.1.6.12; Kalyaanamoorthy et al. 2017; Nguyen et al. 2015). The newly sequenced mitogenome of *C. opalescens* can be accessed at GenBank (OK638184). The complete mitogenome is 23,411 bp long showing the expected gene composition and arrangement: 13 PCGs, 22 transfer RNA, 2 ribosomal RNA genes, with 14 tRNA, 2 rRNA all PCG (except NAD6) being present in the heavy strand (Satoh et al. 2016). We were able to detect and assemble the Holocephali-specific long noncoding insertion present between the tRNAThr and tRNAPro (Inoue et al. 2010).

The phylogeny (Figure 1) is divided into two main subclasses: the Holocephali and the Elasmobranchii, reciprocally monophyletic (Boisvert et al. 2019).

Within the Holocephali, there are three well-supported clades, Chimaeridae, Rhinochimaeridae and Callorhinchidae (Arnason et al. 2001; Inoue et al. 2010). As expected, *C. opalescens* is placed within the family Chimaeridae. However, as previously observed (Gomes-dos-Santos et al. 2020, 2021), neither *Hydrolagus* nor *Chimera* genera were recovered as monophyletic, which highlights the importance of revising the taxonomy. Indeed, previous authors had already suggested that the distinction between *Chimera* and *Hydrolagus* based on the presence or absence of a notch separating the anal from the caudal fin, respectively, needed revision (Didier et al. 2012 and references therein).

**Figure 1.**
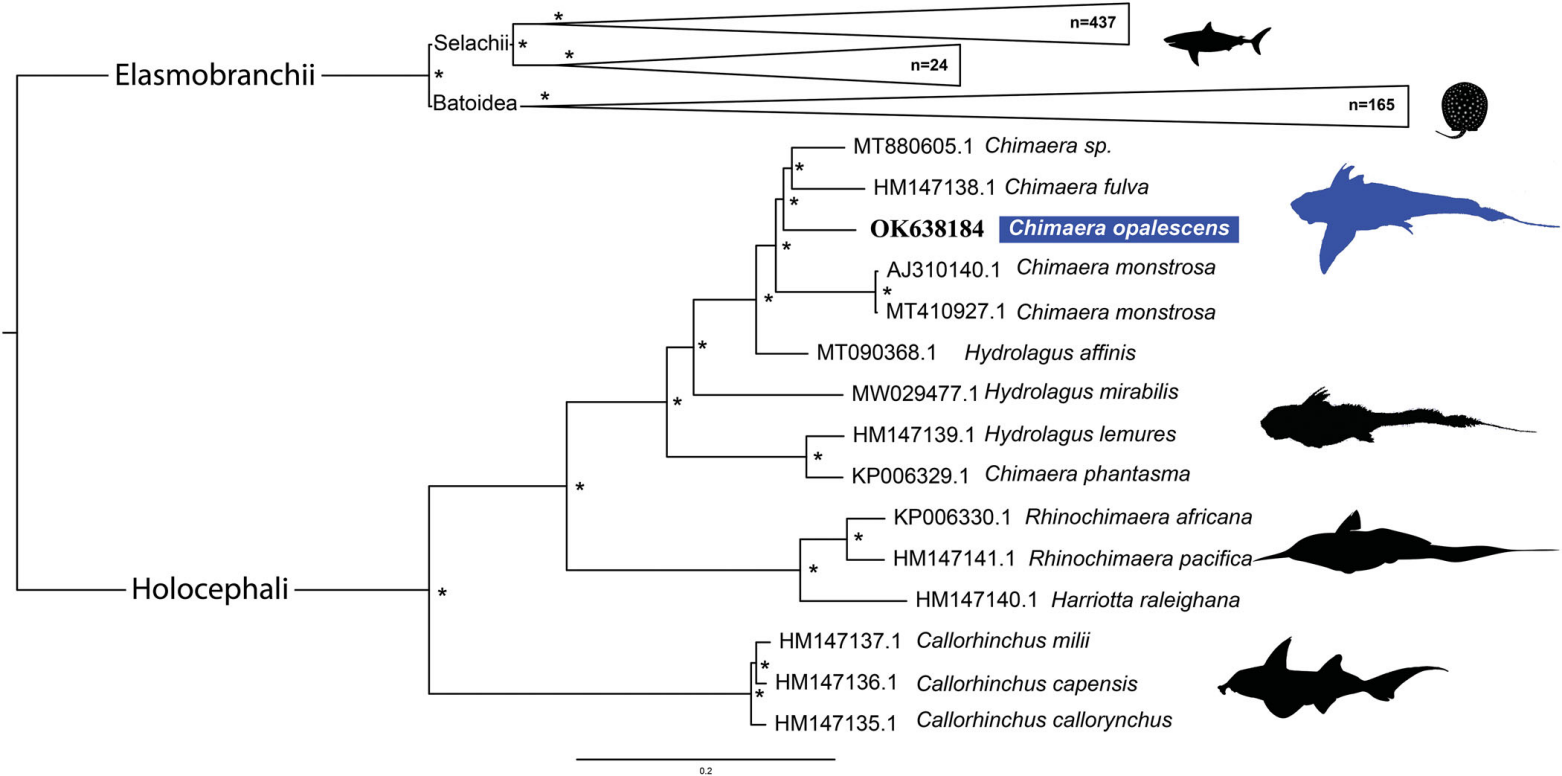
Maximum likelihood phylogenetic tree based on concatenated sequences of 13 protein-coding genes from 641 Chondrichthyan mitogenomes. GenBank accession numbers are listed before species names. The * above the branches indicates that bootstrap support values are above 95%.

## Data Availability

The genome sequence data that support the findings of this study are openly available in GenBank of NCBI at [https://www.ncbi.nlm.nih.gov] under the accession number OK638184. The associated BioProject, SRA, and Bio-Sample numbers are PRJNA778622, SRR16846874 and SAMN22967859 respectively.
